# Alzheimer’s Disease Diagnosis: Discrepancy between Clinical, Neuroimaging, and Cerebrospinal Fluid Biomarkers Criteria in an Italian Cohort of Geriatric Outpatients: A Retrospective Cross-sectional Study

**DOI:** 10.3389/fmed.2017.00203

**Published:** 2017-11-22

**Authors:** Giulia A. M. Dolci, Sarah Damanti, Valeria Scortichini, Alessandro Galli, Paolo D. Rossi, Carlo Abbate, Beatrice Arosio, Daniela Mari, Andrea Arighi, Giorgio G. Fumagalli, Elio Scarpini, Silvia Inglese, Maura Marcucci

**Affiliations:** ^1^Geriatric Unit, Fondazione IRCCS Ca’ Granda Ospedale Maggiore Policlinico, Milan, Italy; ^2^Department of Clinical Sciences and Community Health, University of Milan, Milan, Italy; ^3^Nutritional Sciences, Università degli Studi di Milano, Milan, Italy; ^4^Geriatric Unit, Medical Department Maggiore Hospital, Bologna, Italy; ^5^Geriatric Unit, ASST Lariana, Ospedale Sant’Anna, Como, Italy; ^6^Neurodegenerative Disease Unit, Department of Pathophysiology and Transplantation, Centro Dino Ferrari, University of Milan, Fondazione IRCCS Ca’ Granda Ospedale Maggiore Policlinico, Milan, Italy; ^7^Department of Neurosciences, Psychology, Drug Research and Child Health (NEUROFARBA), University of Florence, Florence, Italy; ^8^Department of Health Research Methods, Evidence, and Impact, McMaster University, Hamilton, ON, Canada

**Keywords:** Alzheimer, aging, clinical criteria, biomarkers, neuropsychological tests, cerebrovascular disease

## Abstract

**Background:**

The role of cerebrospinal fluid (CSF) biomarkers, and neuroimaging in the diagnostic process of Alzheimer’s disease (AD) is not clear, in particular in the older patients.

**Objective:**

The aim of this study was to compare the clinical diagnosis of AD with CSF biomarkers and with cerebrovascular damage at neuroimaging in a cohort of geriatric patients.

**Methods:**

Retrospective analysis of medical records of ≥65-year-old patients with cognitive impairment referred to an Italian geriatric outpatient clinic, for whom the CSF concentration of amyloid-β (Aβ), total Tau (Tau), and phosphorylated Tau (p-Tau) was available. Clinical diagnosis (no dementia, possible and probable AD) was based on the following two sets of criteria: (1) the *Diagnostic Statistical Manual of Mental Disorders* (*DSM*-IV) plus the National Institute of Neurological and Communicative Disorders and Stroke and the Alzheimer’s Disease and Related Disorders Association (NINCDS-ADRDA) and (2) the National Institute on Aging-Alzheimer’s Association (NIA-AA). The Fazekas visual scale was applied when a magnetic resonance imaging scan was available.

**Results:**

We included 94 patients, mean age 77.7 years, mean Mini Mental State Examination score 23.9. The concordance (kappa coefficient) between the two sets of clinical criteria was 70%. The mean CSF concentration (pg/ml) (±SD) of biomarkers was as follows: Aβ 687 (±318), Tau 492 (±515), and p-Tau 63 (±56). There was a trend for lower Aβ and higher Tau levels from the no dementia to the probable AD group. The percentage of *abnormal liquor* according to the local cutoffs was still 15 and 21% in patients without AD based on the *DSM*-IV plus NINCDS-ADRDA or the NIA-AA criteria, respectively. The exclusion of patient in whom normotensive hydrocephalus was suspected did not change these findings. A total of 80% of patients had the neuroimaging report describing chronic cerebrovascular damage, while the Fazekas scale was positive in 45% of patients overall, in 1/2 of no dementia or possible AD patients, and in about 1/3 of probable AD patients, with no difference across ages.

**Conclusion:**

We confirmed the expected discrepancy between different approaches to the diagnosis of AD in a geriatric cohort of patients with cognitive impairment. Further research is needed to understand how to interpret this discrepancy and provide clinicians with practical guidelines.

## Introduction

For decades, Alzheimer’s disease (AD) has been diagnosed only based on clinical criteria (1). With the increasing knowledge of the pathogenic processes underlying this and other dementias, several biomarkers have been proposed to support the diagnosis, also at early stages (2–4). Biomarkers are defined as any objective measurement of an *in vivo* pathological process (5). Cerebrospinal fluid (CSF) proteins [amyloid-β (Aβ) protein, total Tau (Tau), and phosphorylated Tau (p-Tau)] and functional and anatomical neuroimaging findings represent the most studied biomarkers of AD.

The role of these biomarkers in the diagnosis of AD in clinical practice has not been completely clarified yet, as both the American (1) and International Working Group Guidelines (6) underline. Understanding their role in the diagnostic workflow might be particularly challenging in an older population (>85 years) presenting with cognitive impairment. There is evidence suggesting that, as age increases, the prevalence of pathological patterns that have been associated with the disease, increases also in subjects without cognitive impairment (7, 8). Also, the association between the presence of neuritic plaques in autoptic specimens and dementia is less strong in older people (9, 10). Changes in neuroimaging may be less salient in the older ages, in which atrophy often coexists with cerebrovascular damage. Furthermore, chronic cerebrovascular disease is such a frequent neuroimaging finding that its contribution to the cognitive deficit remains difficult to define, especially when not properly quantified (11). Finally, in the oldest patients, the burden of comorbidities often makes the scenario more complex (6). All these reasons increase the chance of conflicting findings between biomarkers and clinical symptoms. The whole picture is further complicated by the fact that the existing sets of diagnostic criteria for AD proposed by different scientific societies assign a different place to some clinical symptoms and signs. In fact, the diagnostic criteria have changed over time, integrating the new knowledge upon the disease mechanisms and biomarkers and reflecting different disease definitions (1, 6, 12). However, the newer criteria have not replaced the older ones, which are still being used in clinical research, and in particular in studies evaluating therapies for AD (13). The dimension of the problem can be substantial and represents a barrier to a straightforward diagnostic process in routine practice, especially in those clinical settings providing care to less selected older patients such as geriatrics.

With such a background, the objective of our study was to represent the level of discrepancy between different diagnostic approaches, describing a population of older patients with cognitive impairment referred to an Italian geriatric outpatient clinic. In particular, we compared the diagnosis based on clinical criteria with the CSF biomarkers and with cerebrovascular damage finding at neuroimaging.

## Materials and Methods

### Study Design and Population

This is a retrospective cross-sectional study of medical records of 65-year-old or older patients, referred to the Alzheimer Evaluation Unit (UVA) of the Division of Geriatrics of the IRCCS Ca’ Granda Ospedale Maggiore Policlinico in Milan between June 2009 and October 2014. Ethical approval was not required for this study in accordance with the institutional guidelines.

We included in the study all those patients with a cognitive impairment who underwent at physician’s discretion a lumbar puncture during the diagnostic workup and for whom the concentration of Aβ, Tau, and p-Tau in the CSF was available. There was no exclusion criterion. In particular, as per our practice, patients undergo a lumbar puncture with liquor collection and examination: (i) in the context of differential diagnosis of dementia, when the treating physician deems it as necessary to help confirm or rule out a clinical suspicion of AD, and (ii) in the context of diagnosis and therapy (i.e., *ex juvantibus*) of normotensive hydrocephalus.

All patients undergo the lumbar puncture only if a specific written informed consent was provided by the patient or by her/his next of kin.

Retrospectively, but in a blind fashion with respect to the actual diagnosis made by the treating physician, we characterized the patients according to different diagnostic approaches. We first classified the patients using two different sets of clinical diagnostic criteria for AD (not taking into account the laboratory findings) and then compared the clinical diagnoses with the results of CSF biomarkers. Second, we described the prevalence of signs of vascular damage at neuroimaging according to different approaches, i.e., standard descriptive reports versus visual quantitative scales, and its correlation with the clinical and the liquor-based classifications. Within this framework, we also evaluated the contribution of neuropsychological (NPS) tests in making the diagnosis of AD. In fact, it is known that many patients with cognitive impairment have poor awareness or understanding of their cognitive impairment (14). Thus, an objective cognitive assessment lies at the core of an appropriate diagnostic workup for cognitive decline. Moreover, NPS tests can help define early or prodromal states like a mild cognitive impairment (MCI), in which biomarkers might be already positive (6, 15, 16). Finally, all the patients for whom a brain magnetic resonance imaging scan was available were included in the sub-study on neuroimaging.

To have an objective comparison across different diagnostic tools, we included all patients with a CSF record, regardless of the final diagnosis made by the treating physician. Patients with a clinical suspicion of normotensive hydrocephalus were included in the main analyses as expected negative cases (i.e., cases in which CSF biomarkers were expected to be negative). They were then excluded as a sensitivity analysis.

### Data Collection

Patient medical records temporarily close but preceding the time of the lumbar puncture were evaluated for the purpose of our study. The study investigators were guarantor for protecting the confidential data from any inappropriate use beyond the purpose of this study.

#### Clinical Diagnostic Criteria and NPS Assessment

Two investigators (GD and AG) screened the patient charts independently and retrospectively reanalyzed medical records of patients included in the study, being blinded to the diagnosis that was made by the treating geriatrician. Clinical diagnosis of dementia and of AD was based on the criteria of the *Diagnostic Statistical Manual of Mental Disorders* (*DSM*-IV) (17) and of the National Institute of Neurological and Communicative Disorders and Stroke and the Alzheimer’s Disease and Related Disorders Association (NINCDS-ADRDA) 1984 (18), respectively (Appendix in Supplementary Material). Patients were also classified according to the criteria for dementia and AD of the National Institute on Aging-Alzheimer’s Association (NIA-AA) 2011 (1) (Appendix in Supplementary Material). Based on each of the two sets of clinical criteria, patients were classified into no dementia, possible AD, and probable AD. The results of the following clinical investigations were taken into account for the classification of each patient upon those criteria, when available: the multidimensional geriatric assessment, blood tests, NPS tests, and neuroimaging. In particular, we relied on the results of the NPS assessment, when available, in case of inconsistency between the NPS report and the record of the geriatric visit for what concerned the presence of memory deficits and the level of impact on function. In order to preserve the comparative analyses of our sub-study, when we applied the clinical diagnostic criteria, we used the neuroimaging reports only to rule out the presence of a clear alternative diagnosis (i.e., normotensive hydrocephalus, multi-infarct disease, and tumor). The descriptive finding of “leukoaraiosis” or “chronic cerebrovascular disease” was not considered sufficient for meeting the criterion of an alternative (i.e., vascular) etiology of dementia.

We looked for the NPS assessment that preceded or coincided with the date of the lumbar puncture. In patients with multiple assessments, we used the outcome of the assessment that was temporarily closer to the date of the lumbar puncture. We used the outcome of NPS tests performed after the lumbur puncture only if temporarily very close (no more than 1 month later). The battery of NPS tests was administered by an expert neuropsychologist. Global cognitive functioning was assessed by means of the Mini Mental State Examination (MMSE) (19) and general intellectual functioning was investigated by using Raven’s colored progressive matrices (20). For temporal orientation, the first item of the MMSE was considered. Anterograde long-term memory was rated with the prose recall test (21) and the delayed recall of the Rey–Osterreith complex figure test (17). Verbal short-term memory was assessed by means of the forward digit span test (18). The digit cancelation test (21) was administered to examine visual attention. Executive prefrontal functions were evaluated using the backward digit span test (17), the trail-making test (22), and the phonological fluency test (23). Spatial skills were divided into spatial orientation, assessed by the second item of the MMSE, and spatial abilities, explored by means of the copy of geometrical figure test (20) and the copy of the Rey–Osterreith complex figure test (17). Language was examined using the picture-naming test (24). All tests, excepted for the orientation one, have been validated and standardized in a sample of healthy Italian subjects. Most of the normative data are referred to the study from Spinnler and Tognoni (21). According to the outcome of the NPS assessment, patients were classified into: normal cognition, MCI, or diffuse/severe cognitive impairment. Patients classified as MCI were divided into the following four subtypes: only memory domain affected, single-domain MCI with a deficit other than on memory domain, multiple domain MCI with memory domain affected, and multiple domain MCI with deficits other than on memory domain.

#### CSF Biomarkers

The lumbar puncture was performed according to procedural standards. The dosage of Aβ protein, Tau, and p-Tau in the liquor was performed on site. The cerebrospinal fluid sample was centrifuged at 4°C and stored at −30°C until analysis. Aβ42 protein, Tau and p-Tau 181 were determined by ELISA kits (Innogenetics). The local laboratory cutoff points for normal protein concentrations are as follows: Aβ42 > 600 pg/ml, Tau < 500 pg/ml, and p-Tau < 61 pg/ml.

#### Neuroimaging

Available brain MRI images were examined independently and retrospectively by three operators blinded to the patient clinical history. The Fazekas visual scale (25) was applied on at least one long TR sequence, Flair or T2. Given the smallest number of missing data, for the purpose of the analysis, only axial plane images were considered. The Fazekas scores range from 0 (normal) to 3 (extensive, diffuse, and confluent lesions of the subcortical white matter). For the purpose of our analyses, we dichotomized the Fazekas scores into negative (0 or 1) and positive (≥2). We chose this cutoff in order to be more specific in this population at high prevalence of chronic cerebrovascular damage.

We decided not to include the assessment of atrophy according to the qualitative versus quantitative approach in our comparative analyses, because only a very small subgroup of patients had suitable MRI images to apply atrophy quantitative scales. Functional neuroimaging (positron emission tomography) was available only for few patients.

### Statistical Analysis

Descriptive statistics (mean, SD, median and range in case of numerical variables, and frequency in case of categorical variables) were used to present the classification of patients according to the clinical criteria (*DSM*-IV plus NINCDS-ADRDA versus NIA-AA), the CSF biomarkers, and the neuroimaging biomarkers of cerebrovascular disease. First, the two different clinical criteria were compared and the concordance was measured by Cohen’s kappa calculation (to take into account the effect of chance). Distributions of biomarkers were compared with the clinical diagnoses according to the NIA-AA criteria, using cross tabulations and Pearson χ^2^ or Kruskal–Wallis test, in the whole cohort and by age groups. As sensitivity analyses, the comparison was repeated (i) excluding patients that underwent the lumber puncture in the context of a suspicion of normotensive hydrocephalus and (ii) taking into account the NPS diagnosis. Inter-rater reproducibility for the MRI visual scales was also calculated as Kappa.

## Results

The clinical records of 94 patients were examined. Table 1 shows the baseline characteristics of the study cohort. In most of the cases (68%), the lumbar puncture was performed in the context of a differential diagnosis for AD. In 11 of these 64 patients, alternative dementia etiologies were considered: Lewi Body Dementia in four patients; Fronto-Temporal Dementia in six patients (in one of these patients normotensive hydrocephalus etiology was also under consideration); and subclinical hypothyroidism in one patient. In 30 patients, normotensive hydrocephalus was the main diagnostic hypothesis and the main reason for the lumbar puncture.

**Table 1:** Baseline characteristics.

Characteristics	Distribution
Mean age (SD), years	77.7 (5.2)
Female, *n* (%)	58 (61.7)
Mean MMSE (SD)	23.9 (4.1)
Mean basic ADL score (SD)	4.7 (1.6)^a^
Mean instrumental ADL score (SD)	4.3 (2.5)^a,b^
History of hypertension, *n* (%)	58 (61.7)
History of diabetes mellitus, *n* (%)	18 (19.1)
History of dyslipidemia, *n* (%)	40 (42.5)
Smoker, *n* (%)	
Yes	53 (53.4)
No	10 (10.6)
Ex	31 (33.0)
History of coronary artery disease, *n* (%)	9 (9.6)
History of stroke or TIA, *n* (%)	8 (8.5)
History of peripheral artery disease, *n* (%)	4 (4.2)
Carotid atherosclerosis, *n* (%)	43 (45.7)^c^

*n, number; MMSE, Mini Mental State Examination; ADL, activity of daily living; TIA, transient ischemic attack*.

*^a^Information missing for one patient*.

*^b^In 35 patients (30 men), The maximum number of applicable items was less than 8*.

*^c^Stenosis of at least 20% at the US scan*.

Table 2 summaries the availability of data for the comparison of the different diagnostic tools.

**Table 2:** Availability of data on the different diagnostic approaches in the study cohort.

Diagnostic approach	Number of patients with data (% of the total cohort)
Clinical criteria	94 (100)
CSF biomarkers	94 (100)
NPS assessment	71 (75)
Neuroimaging—standard report	76 (81)
Neuroimaging—Fazekas scale	40 (42)

*CSF, cerebral spinal Cerebrospinal fluid; NPS, neuropsychological*.

### Classifications According to Clinical Diagnostic Criteria and CSF Biomarkers

A total of 55 (58%), 13 (14%), and 26 (28%) patients were classified as being affected by no dementia, possible AD, and probable AD, respectively, according to the *DSM*-IV plus NINCDS-ADRDA criteria; 39 (41%), 27 (29%), and 28 (30%), respectively, according to the NIA-AA. As pre-specified, criterion on the presence of memory deficits was fulfilled using the objective outcome of the NPS assessment. In fact, 64% of those patients who had no objective memory deficit at the NPS tests had expressed a memory complaint during the clinical visit.

Table 3 compares patient classification according to the *DSM*-IV plus NINCDS-ADRDA with the NIA-AA clinical criteria. Every patient who was classified as demented according to the *DSM*-IV criteria was also classified as demented according to the NIA-AA criteria; whereas 29% classified as demented according to the NIA-AA criteria were not demented according to *DSM*-IV criteria. The crude concordance between the two sets of criteria for the diagnosis of dementia was 83%, with a kappa coefficient of 67%. The crude concordance for the specific diagnosis (no dementia, possible and probable AD) between the two criteria was 81% with a kappa of 70%.

**Table 3:** Comparison of *Diagnostic Statistical Manual of Mental Disorders* (*DSM*-IV) plus National Institute of Neurological and Communicative Disorders and Stroke and the Alzheimer’s Disease and Related Disorders Association (NINCDS-ADRDA) and National Institute on Aging-Alzheimer’s Association (NIA-AA) criteria for the diagnosis of Alzheimer’s disease (AD).

Clinical diagnostic criteria		NIA-AA criteria			
		No dementia, *n* (%)	Possible AD, *n* (%)	Probable AD, *n* (%)	Total, *n* (%)
*DSM*-IV plus NINCDS-ADRDA criteria	No dementia, *n* (%)	39 (71)	16 (29)	0 (0)	55 (58)

	Possible AD, *n* (%)	0 (0)	11 (85)	2 (15)	13 (14)

	Probable AD, *n* (%)	0 (0)	0 (0)	26 (100)	26 (28)

	Total, *n* (%)	39 (41)	27 (29)	28 (30)	94 (100)

The mean (SD) value of Aβ, Tau, and p-Tau in the study population was 687 pg/ml (318), 492 pg/ml (515), and 63 pg/ml (56), respectively. According to the local laboratory cutoffs, Aβ and Tau values were on average normal whereas mean p-Tau values were abnormal (high).

**Figure 1:**
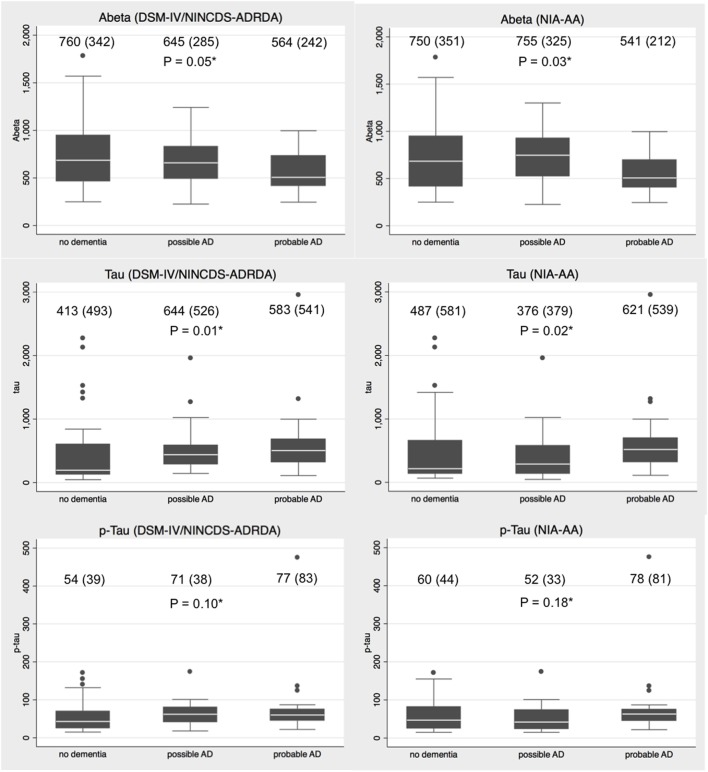
Distributions of amyloid-β (Aβ), total Tau (Tau), and phosphorylated Tau (p-Tau) values in no dementia, possible Alzheimer’s disease (AD) and probable AD patients according to *Diagnostic Statistical Manual of Mental Disorders* (*DSM*-IV) plus National Institute of Neurological and Communicative Disorders and Stroke and the Alzheimer’s Disease and Related Disorders Association (NINCDS-ADRDA) and National Institute on Aging-Alzheimer’s Association (NIA-AA) criteria. Legend: mean (SD) concentration is provided for each diagnostic category. *Kruskal–Wallis test for difference in the protein distribution across diagnostic groups.

There was a statistically significant difference in the CSF concentration of Aβ and Tau but not of p-Tau, across the three diagnoses made according to both NINCDS-ADRDA and NIA-AA criteria (Figure 1). In particular, there was a trend for lower Aβ values and higher Tau levels going from the no dementia group to probable AD group, more evident in the case of the NINCDS-ADRDA diagnoses.

When the biomarkers levels were dichotomized based on local lab cutoffs into *positive* (i.e., abnormal) or *negative* (i.e., normal), the frequency of biomarkers positivity differed across the diagnoses in a statistically significant way only for Aβ, with both *DSM*-IV plus NINCDS-ADRDA and NIA-AA classification (4). Every biomarker tended to be more frequently positive in the case of patients with a diagnosis of probable AD compared to patients with a diagnosis of possible AD or no dementia (Table 4). Compared to patients with no dementia, patients with possible AD tended to present with positive biomarkers more frequently when *DSM*-IV plus NINCDS-ADRDA criteria were used but less frequently when NIA-AA criteria were used (Table 4).

**Table 4:** Relationship between CSF biomarkers and clinical diagnosis.

	*Diagnostic Statistical Manual of Mental Disorders*-IV plus National Institute of Neurological and Communicative Disorders and Stroke and the Alzheimer*’*s Disease and Related Disorders Association criteria			National Institute on Aging*-* Alzheimer*’*s Association criteria		
	Amyloid-β (Aβ)	Total tau (Tau)	Phosphorylated tau (p-Tau)	Aβ	Tau	p-Tau
		
	Positive, *n* (%)	Positive, *n* (%)	Positive, *n* (%)	Positive, *n* (%)	Positive, *n* (%)	Positive, *n* (%)
No dementia	20 (36)	17 (31)	19 (35)	16 (41)	12 (31)	15 (39)
Possible AD	5 (38)	5 (38)	7 (54)	8 (30)	8 (30)	9 (33)
Probable AD	18 (69)	13 (50)	13 (50)	19 (68)	15 (54)	15 (54)
Pearson χ^2^, *p*	**0.012**	0.251	0.261	**0.013**	0.102	0.277

Then, the CSF biomarkers were considered as a whole and the patient classified as having *positive liquor* only when the level of all the three proteins was abnormal (i.e., reduced Aβ, elevated Tau, and p-Tau). In this case, only 18 patients (19%) had *positive liquor*. Patients with *positive liquor* were on average younger than those with *negative liquor* [mean age 74.7 (SD ±3.7) versus 77.2 (SD ±2.7), *p* for Kruskal–Wallis test = 0.002].

Table 5 shows the distribution of the *liquor* biomarker according to the different diagnoses and to different age groups. The trend for *positive liquor* was the same in the whole population and in the two age groups, with a higher prevalence of *positive liquor* in probable AD than in possible AD and no dementia, in both clinical classifications. The prevalence was again higher in those with no dementia than in those with possible dementia in the case of NIA-AA criteria. In any age group and in any clinical diagnosis group, a *negative liquor* was more prevalent than *positive liquor* (Table 5).

**Table 5:** Distribution of the biomarker *liquor* according to the *Diagnostic Statistical Manual of Mental Disorders* (*DSM*-IV) plus National Institute of Neurological and Communicative Disorders and Stroke and the Alzheimer’s Disease and Related Disorders Association (NINCDS-ADDRDA) and National Institute on Aging-Alzheimer’s Association (NIA-AA) diagnosis and age groups.

Age (years)	*DSM*-IV plus NINCDS-ADRDA criteria			NIA-AA criteria		
	Diagnosis	Positive liquor, *n* (%)	Pearson χ^2^, *p*	Diagnosis	Positive liquor, *n* (%)	Pearson χ^2^, *p*
Any	No dementia	8 (15)	0.208	No dementia	8 (21)	**0.027**
	Possible AD	2 (15)		Possible AD	2 (4)	
	Probable AD	8 (31)		Probable AD	8 (32)	

≥80	No dementia	2 (7)	0.324	No dementia	2 (11)	0.321
	Possible AD	0 (0)		Possible AD	0 (0)	
	Probable AD	1 (20)		Probable AD	1 (20)	

<80	No dementia	6 (24)	0.494	No dementia	6 (27)	0.168
	Possible AD	2 (16)		Possible AD	1 (7)	
	Probable AD	7 (33)		Probable AD	8 (35)	

When we considered age cutoffs progressively lower than 80, the percentage of patients with *positive liquor* became higher than the percentage of patients with *negative liquor* only among patients with a diagnosis of probable AD (any set of criteria) younger than 76 years (66% positive versus 33% negative).

Thirty of the 94 patients underwent a lumbar puncture in the context of a clinical suspicion of normotensive hydrocephalus. When re-classified in a blinded fashion according to the two sets of clinical criteria, these patients were all classified as with no dementia according to the *DSM*-IV criteria. According to the NIA-AA criteria, 19 (63%) patients were not demented and 11 (27%) patients had a possible AD. Table 6 shows the comparison between the clinical (NIA-AA criteria) and the liquor diagnoses when patients with normotensive hydrocephalus were excluded. The trend did not change compared with the main analysis.

**Table 6:** Distribution of the biomarker *liquor* according to National Institute on Aging-Alzheimer’s Association (NIA-AA) diagnosis in patients without a clinical suspicion of normotensive hydrocephalus.

Diagnosis NIA-AA criteria	Positive liquor, *n* (%)	Pearson χ^2^, *p*
No dementia	7 (35)	**0.102**
Possible AD	1 (4)	
Probable AD	9 (32)	
Any	17 (27)	

### NPS Assessment

The outcome of the NPS assessment was available for 71 patients. Only one patient had a normal test performance; 44 patients were diagnosed as affected by diffuse cognitive impairment; and 26 patients were diagnosed as affected by MCI. Age was no significantly different between patients with diffuse cognitive impairment (mean 76.4 years, SD ±3.8) and patients with MCI (mean 76.4 years SD ±5.5). Twenty-six of the 27 patients classified as with no dementia according to the NIA-AA criteria (96.3%) were diagnosed as affected by MCI, most of them (88%) with deficits in multiple cognitive domains. In particular, the definition into MCI subtypes was available for 25 patients: three (12%) patients were classified as single MCI with only memory domain affected; 15 (60%) patients were classified as multiple domain MCI with memory domain affected; and seven (26%) patients were classified as multiple domain MCI with deficits other than on memory domain. No patient was classified as single-domain MCI with a deficit other than on memory domain.

Tables 7 and 8 show the frequency of liquor positivity according to the NPS outcome and MCI phenotypes, respectively. There was no statistically significant difference in the frequency of positivity across NPS definitions. In particular, the biomarker liquor tended to be more frequently positive in patients with MCI than in patient with diffuse cognitive impairment.

**Table 7:** Positivity of the biomarker according to different neuropsychological (NPS) diagnosis.

	Positive liquor among all patients with NPS assessment (71), *n* (%)	Positive liquor among those patients with NPS assessment with no normotensive hydrocephalus suspect (62), *n* (%)
Cognitive normal	1 (100)	–
Mild cognitive impairment	7 (27)	7 (35)
Diffuse cognitive impairment	10 (23)	10 (24)
Total	18 (25)	17 (27)

**Table 8:** Positivity of the biomarker liquor according to different mild cognitive impairment (MCI) phenotypes and age groups.

Age (years)	Neuropsychological phenotype	Negative liquor, *n* (%)	Positive liquor, *n* (%)
≥80	Amnestic MCI	0	0
	Multiple domain MCI+	3 (60)	2 (40)
	Multiple domain MCI−	5 (100)	0 (0)

<80	Amnestic MCI	2 (67)	1 (33)
	Multiple domain MCI+	7 (70)	3 (30)
	Multiple domain MCI−	1 (50)	1 (50)

*+, with amnestic component; −, without amnestic component*.

### Cerebrovascular Burden at Neuroimaging

MRI images were available for 40 patients. Thirty-two of these 40 (80%) patients had a diagnosis of cerebrovascular damage according to the qualitative report made by the radiologist. Mean Fazekas score was 1.55 ± 1. According to the Fazekas score 18 of the 40 (45%) patients were *positive*. Table 9 shows the mean Fazekas scores and positivity according to the clinical diagnostic criteria. According to both clinical classifications, Fazekas was positive in about half of the patients with no dementia or possible AD, while it was positive in about one-third of the patients with probable AD. When only patients with a diagnosis of probable AD according to the NIA-AA criteria were considered, the proportion of patients with a positive Fazekas in progressively younger subgroups remained the same or increased compared with the whole population or with the oldest ones (Table 10). The results were the same for patients with probable AD according to *DSM*-IV plus NINCDS-ADRDA criteria.

**Table 9:** Cerebrovascular damage at neuroimaging according to the Fazekas scale and clinical diagnosis of dementia.

Diagnosis	*Diagnostic Statistical Manual of Mental Disorders*-IV plus National Institute of Neurological and Communicative Disorders and Stroke and the Alzheimer*’*s Disease and Related Disorders Association criteria			National Institute on Aging*-*Alzheimer*’*s Association criteria		
	Fazekas, mean (SD)	Positive Fazekas,^a^ *n* (%)	*p* (Pearson χ^2^ test)	Fazekas, mean (SD)	Positive Fazekas,^a^ *n* (%)	*p* (Pearson χ^2^ test)
No dementia	1.1 (1.1)	12 (52)	0.453	1.6 (1.1)	10 (53)	0.451
Possible AD	1.2 (0.9)	2 (50)		1.6 (1.1)	4 (50)	
Probable AD	1.4 (1.0)	4 (31)		1.3 (1.0)	4 (31)	

*^a^Score ≥2*.

**Table 10:** Distribution of the positive Fazekas score in different age subgroups in people with a clinical diagnosis of probable AD (National Institute on Aging-Alzheimer’s Association criteria).

Age (years)	Positive Fazekas, *n* (%)
≥80	1 (33)
<80	3 (30)
<75	2 (40)
<72	1 (100)

When in the same patients with a clinical diagnosis of probable AD (according to NIA-AA or NINCDS-ADRDA criteria) the Fazekas results were cross-tabulated with the CSF results (Table 11), there was no statistically significant difference in frequency distribution.

**Table 11:** Correlation of the biomarker liquor and cerebrovascular burden at neuroimaging according to Fazekas scores, in subjects with a clinical diagnosis of probable AD (National Institute on Aging-Alzheimer’s Association criteria) in different age subgroups.

	Negative Fazekas, *n* (%)	Positive Fazekas, *n* (%)	*p*
**Total**			
Negative liquor	6 (67)	3 (33)	0.764
Positive liquor	3 (75)	1 (25)	
**<80 years**			
Negative liquor	4 (67)	2 (37)	0.778
Positive liquor	3 (75)	1 (25)	
**≥80 years**			
Negative liquor	2 (67)	1 (33)	–
Positive liquor	0 (0)	0 (0)	
**≤75 years**			
Negative liquor	1 (50)	1 (50)	0.709
Positive liquor	2 (67)	1 (33)	
**>75 years**			
Negative liquor	5 (71)	2 (29)	0.537
Positive liquor	1 (100)	0 (0)	

## Discussion

Our retrospective analysis of a cohort of patients with a cognitive deficit referring to a geriatric outpatient clinic (mean age 78 years), confirmed a non-negligible discrepancy between the diagnosis of AD when based on clinical criteria, CSF biomarkers, or neuroimaging.

First, we confirmed a substantial discordance between the two sets of clinical diagnostic criteria, i.e., *DSM*-IV plus NINCDS-ADRDA (1984) versus NIA-AA (2011) criteria, with an agreement of only 70% when adjusted for the effect of chance. The discordance likely reflects the evolution in the definition of dementia and AD and was someway expected. However, we wanted to quantify this discrepancy in a cohort of patients with a higher probability of a complex phenotype, since 1984 criteria have been used to define patient eligibility for approval studies of many current drugs available for AD and are still being used in research (13, 26–29). According to our data, most (16 out of 27, 59%) of the “possible AD” patients according to the newer criteria would have been classified as “no dementia” by the older approach (Table 3) and would have been not eligible for those studies. The results of those studies are therefore not necessarily applicable to this subset of patients defined as “possible AD” according to a more comprehensive understanding of the disease.

One of the main differences between the two criteria is that the older ones, but not the newer, include the presence of amnesic deficits as a necessary criterion for AD diagnosis. Interestingly, in our cohort, the majority of patients (64%), who had no objective memory deficiency at the NPS tests, had in fact complained about forgetfulness during the clinical visit. This datum has been already described in the literature (30). This confirmed the role of the NPS assessment, which in practice might be sometimes forgone in the assessment of the oldest old patients. An extended NPS battery helped us to better define not only the phenotype but also the severity of the cognitive disorder (6, 31, 32), which, in some cases, allowed us to suppose a higher functional impairment, or a higher contribution of the cognitive deficits to the functional impairment, among other possible health and social contributors, compared to what the interview with the patients or their caregivers had suggested.

The distribution of the CSF biomarkers levels in our population was quite sparse, even in patients with a clinically probable AD. In particular, patients with positive liquor biomarkers still represented a minority among those that would have been classified as probable AD based on clinical criteria only; they represented the majority only in a younger (i.e., <76 years) subset of patients. In 2012, Mattsson et al. investigated the effect of age on the diagnostic performance of CSF biomarkers in a large multi-center study population and they found that although the diagnostic accuracy for AD decreased with age, the predictive values for a combination of biomarkers remained essentially stable. In comparison with our population, their cross-sectional cohort of patients with AD had a lower median age (71 versus 77.7), an higher percentage of male subjects (57 versus 38.3%) and a lower MMSE median score (22 versus 23.9) (33). In that study the clinical diagnosis of AD was based on *DSM*-IV plus NINCDS-ADRDA criteria. In our study too, there was a non-statistically significant trend for an increased liquor positivity going from “no dementia” to “possible” and then “probable AD,” only when the *DSM*-IV plus NINCDS-ADRDA were used. This suggests a higher concordance between the CSF biomarkers so far known and the classical AD variant, rather than with the more comprehensive AD definition. In contrast, when we looked at the relationship between the CSF protein distribution and the NPS outcome, we did not find the expected association between a classical amnesic MCI phenotype and positive biomarkers.

Our findings confirm that quantitative methods based on neuroimaging (i.e., the Fazekas scale) can help refine the classification of patients upon the degree of cerebrovascular damage compared to descriptive radiological reports (34). Yet, the clinical relevance of neuroimaging remains uncertain among relatively older patients. Indeed, we less frequently found a positive Fazekas in patients with probable AD, compared with patient with possible or no AD, suggesting that the vascular damage is not a typical pathogenic mechanism of the disease. However, there was still a substantial percentage (31%) of positive Fazekas among patients with probable AD. Furthermore, we found that a positive Fazekas tended to be only slightly more frequent among probable AD with *negative liquor* than among those with a *positive liquor*, regardless of age. This finally suggests that the two pathogenic pathways, i.e., the vascular and the degenerative, can definitely coexist and not necessarily only in patients who would easily meet the definition of vascular/mixed dementia (such as patients with a history of stroke).

The retrospective nature and the small sample size are the main limitations of our study, which could be only descriptive and explorative in nature. Then, although less selected in terms of age and clinical complexity than in randomized controlled trials, our cohort still represented a selected population. Indeed, including only patients who underwent a lumbar puncture might have led to the exclusion of the oldest and most complex patients for whom the lumbar puncture is more frequently thought not to have a favorable risk-benefit profile. Finally, we had to deal with missing and incomplete information, given the retrospective nature of our study and the use of data from routine practice. The time lag between the date in which the patient underwent the lumbar puncture and the time in which some other study variables were collected could represent a limitation to the actual concurrency of the cross-sectional comparison. However, this is consistent with the routine practice.

## Conclusion

To conclude, we showed a significant degree of discordance between clinical criteria, NPS assessment, liquor biomarkers, and neuroimaging when used to characterize cognitive disorders in geriatric outpatients. Given the methodological limitations of our study, prospective larger multi-center studies, including inception cohorts of unselected patients that undergo a clinical, laboratory, and neuroradiological assessment, and with a clinical follow-up, would be theoretically necessary to better understand the role of biomarkers in the diagnostic workup of geriatric patients with cognitive disorders. However, practical and ethical issues might hinder the conduction of such a type of study, while the current demographic trend will lead quickly to a further increase of the prevalence of this patient population. Hence, researchers and clinicians in the field should make the efforts to combine their experience and expertise to reach a consensus on the best diagnostic practice in this population.

## Ethics Statement

The study was a retrospective analyis of data routinely collected for clinical purpose, retrieved from patient medical charts. No approval from an ethics committee was sought. Given the nature of the study, the patients did not provide a specific consent. They had provided an informed consent to the clinical procedures performed as outpatients at the geriatric clinic, as per good clinical practice. The analyses were performed, and the results reported with full respect of anonymity and confidentiality. The data retrieved for the purpose of this study were not used for any other purpose.

## Author Contributions

GD and MM are the lead investigators and headed study design, data collection, and article writing. MM is also the senior mentor of the study and oversaw the study design. VS, AG, AA, and GF are coinvestigators and participated in study design, data collection, and article review. SD helped with statistical analysis and participated in article review. PR, CA, SI, and BA contributed to the study design, facilitated access to medical records, and participated in article review. DM is the medical director of the Geriatric Unit of the Fondazione IRCCS Ca’ Granda Ospedale Maggiore Policlinico in Milan. ES is the responsible of the Alzheimer Evaluation Unit of the Neurodegenerative disease Unit of the Fondazione IRCCS Ca’ Granda Ospedale Maggiore Policlinico in Milan. DM and ES are the guarantors for study compliance to ethical principles and participated in article review. All the authors reviewed and accepted the final version of the article.

## Conflict of Interest Statement

The authors declare that the research was conducted in the absence of any commercial or financial relationships that could be construed as a potential conflict of interest.
