# Supplementary material for: Alzheimer’s Disease Diagnosis: Discrepancy between Clinical, Neuroimaging, and Cerebrospinal Fluid Biomarkers Criteria in an Italian Cohort of Geriatric Outpatients: A Retrospective Cross-sectional Study
Source: Front Med (Lausanne). 2017 Nov 22;4:203. doi: 10.3389/fmed.2017.00203 (PMC5702632; doi:10.3389/fmed.2017.00203)
Supplement: Supplementary file 1 [file data_sheet_1.docx]

**APPENDIX**

Relevant aspects of the DSM-IV and NIA-AA diagnostic criteria for dementia and of the NINCDS-ADRDA and NIA-AA diagnostic criteria for Alzheimer’s disease (1, 20, 21)

|  | **DSM-IV** | **NIA-AA (2011)** |
| --- | --- | --- |
| **Dementia criteria** | Memory impairment and at least one of the following: aphasia, apraxia, agnosia, disturbance in executive functioning | The cognitive or behavioral impairment involves a minimum of two of the following domains: acquire and remember new information, reasoning and handling of complex tasks, visuospatial abilities, language functions, changes in personality/behavior or comportment |
|  | The cognitive deficits cause significant impairment in social or occupational functioning and represent a significant decline from a previous level of functioning.. The cognitive deficits do not occur exclusively during the course of delirium. The disturbance is not better accounted for by another axis I disorder (e.g. major depressive disorder) | Cognitive or behavioral symptoms interfere with the ability to function at work or at usual activities and represent a decline from a previous level of functioning and performing and are not explained by delirium or major psychiatric disorder |
|  | The course is characterized by gradual onset and continuing cognitive decline | Cognitive impairment is detected and diagnosed through a combination of history-taking from the patient and a knowledgeable informant and an objective cognitive assessment |
|  | The cognitive deficits are not due to: other central nervous system conditions, systemic conditions that are known to cause dementia, substance-induced conditions |  |
|  |  |  |
|  | **NINCDS-ADRDA (1984)** | **NIA-AA (2011)** |
| **Dementia criteria** | **Not provided** | **Provides criteria for all-cause dementia** |
| **Probable AD** |  |  |
| **Dementia diagnosis** | Impairments in two cognitive domains based on | Impairments in two cognitive domains, and |
|  | clinical exam and documented by cognitive testing | expands the definition on the non memory forms of AD (language, visuospatial, executive) |
|  |  |  |
| **Onset and progression** | Progressive worsening of memory symptoms and other cognitive functions | Insidious onset and clear-cut history of worsening |
|  |  | of cognition by report or observation |
| **Comorbid systemic or** | Absence of systemic or neurological disorders that in | Absence of cerebrovascular disease or other |
| **neurological disorders** | and of themselves could account for the cognitive deficits | neurological, nonneurological comorbidities or use of medication that could |
|  |  | have substantial effect on cognition |
|  |  |  |
| **Age** | Between ages 40 and 90 | No age limitation |
| **Behavioral and neurological** | Altered pattern of behavior and mood-related | Mood-related and behavioral symptoms are |
| **symptoms** | disorders (e.g. depression), increased muscle tone, myoclonus, gait disorders | considered a ‘domain’ in the definition of dementia |
|  |  |  |
| **Level of consciousness** | The diagnosis of AD cannot be made in patients with | Symptoms cannot be explained by delirium or other major psychiatric disorder |
|  | delirium, drowsiness, stupor/coma, or other |  |
|  | abnormality that prevent adequate evaluation. |  |
| **Laboratory test** | Normal lumbar puncture and blood tests. CT scan normal or with atrophy | Not stated |
|  |  |  |
| **Biomarkers** | Not available in 1984 | MRI, PET, and CSF studies. Any biomarker |
|  |  | positive increases the certainty of AD in patients |
|  |  | with probable AD. Recommended only for research purposes or clinical trials |
|  |  |  |
| **Familial forms** | Familial history of similar disorders, particularly if | Evidence of a causative gene (*APP, PSEN1, and* |
|  | confirmed by autopsy supports the diagnosis of AD | *PSEN2*) increases the likelihood of AD pathology. |
|  |  | The *APOE-4* allele is not sufficiently specific to be considered in this category |
|  |  |  |
| **Possible AD** |  |  |
| **Comorbid conditions** | Presence of systemic or neurological disorders that in | Meets clinical criteria for AD but there is |
|  | and of themselves could account for the cognitive | cerebrovascular disease, or other neurological or |
|  | deficits, which is not considered to be the cause of the | non-neurological comorbidities, or use of |
|  | dementia | medication that could have substantial effect on |
|  |  | cognition |
| **Atypical presentations** | Presence of variations in the presentation, onset, or clinical course | Presence of atypical course, sudden onset, or there |
|  |  | is insufficient historical detail or documentation of progressive decline |
|  |  |  |
| **Single cognitive domain** | Presence of a single cognitive deficit in the absence of other identifiable cause | Replaced by MCI |
|  |  |  |
| **Non-AD phenotype** | Not addressed | At least two biomarker categories positive (Aβ |
|  |  | CSF, tau CSF, PET, or MRI) to support the |
|  |  | presence of underlying AD pathology |
